# Glomus Tumor of the Stomach: Depiction by Multidetector CT and Three-Dimensional Volume Rendering Imaging

**DOI:** 10.1155/2010/126095

**Published:** 2010-03-03

**Authors:** Tarak H. Patel, Karen M. Horton, Ralph H. Hruban, Elliot K. Fishman

**Affiliations:** ^1^Russell H. Morgan Department of Radiology and Radiological Science, Johns Hopkins Hospital, JHOC 3254, 601 North Caroline Street, Baltimore, MD 21287-0801, USA; ^2^Department of Radiology, Johns Hopkins Medical Institutions, 601 N. Caroline Street, JHOC 3253, Baltimore, MD 21287, USA; ^3^Department of Pathology, Johns Hopkins Medical Institutions, Baltimore, MD 21287, USA

## Abstract

Glomus tumors are uncommon tumors which can occur anywhere within the gastrointestinal tract but have been shown to occur most commonly in the gastric antrum. On CT, these tumors demonstrate hyperenhancement which may help distinguish them from other gastric masses.

## 1. Introduction

Glomus tumors originate in the neuromyoarterial glomus, a specialized arteriovenous shunt that is abundantly supplied with nerve fibers to help regulate skin temperature [1]. Glomus tumors are typically found in peripheral soft tissues, such as the dermis or subungual region, but can occur anywhere including the gastrointestinal tract, nerves, nasal cavity, and trachea [1]. Glomus tumors of the gastrointestinal tract are particularly rare and were initially reported in 1951 by Vanwijnsberghe et al. [2]. To date, the largest single study, which was conducted by Miettinen et al. at the Armed Force Institute of Pathology, reported 32 gastrointestinal glomus tumor cases of which 31 were within the stomach and one was located in the cecum [3]. 

We present the case of a biopsy proven gastric glomus tumor evaluated utilizing multidetector computed tomography (MDCT) and multiplanar reconstructions, which to our knowledge have not been previously discussed in the literature. 

## 2. Case Report

A 58-year-old female with a past medical history of gastroesophageal reflux disease refractory to medical therapy underwent a routine esophagogastroduodenoscopy that revealed Barrett's esophagus, gastritis, and a 2 cm × 2 cm submucosal mass in the gastric antrum. Endoscopic resection of the submucosal mass was attempted but the lesion could not be safely removed. Biopsies, however, were obtained. A CT scan was performed to characterize the mass.

Dual phase abdominal CT and 3D CT imaging was performed utilizing a 64 slice MDCT (Sensation 64, Siemens Medical Solutions, Malvern PA) with 0.6 mm collimation. 0.75 mm slices were reconstructed at 0.5 mm intervals for multiplanar reconstructions. 5 mm slices were also reconstructed at 5 mm intervals for review on Picture Archive Computer Systems. Patient was administered 100 cc of intravenous contrast material (Omnipaque 350—GE Healthcare, Princeton NJ), infused at a rate of 3-4 mL/s. Scanning delays were 25 and 50 seconds for imaging of the arterial and venous phases, respectively. Water was administered for oral contrast. The patient was imaged in the supine position. Multiplanar reconstructions were created. Images were reviewed by a radiologist with extensive CT experience.

**Figure 1:**
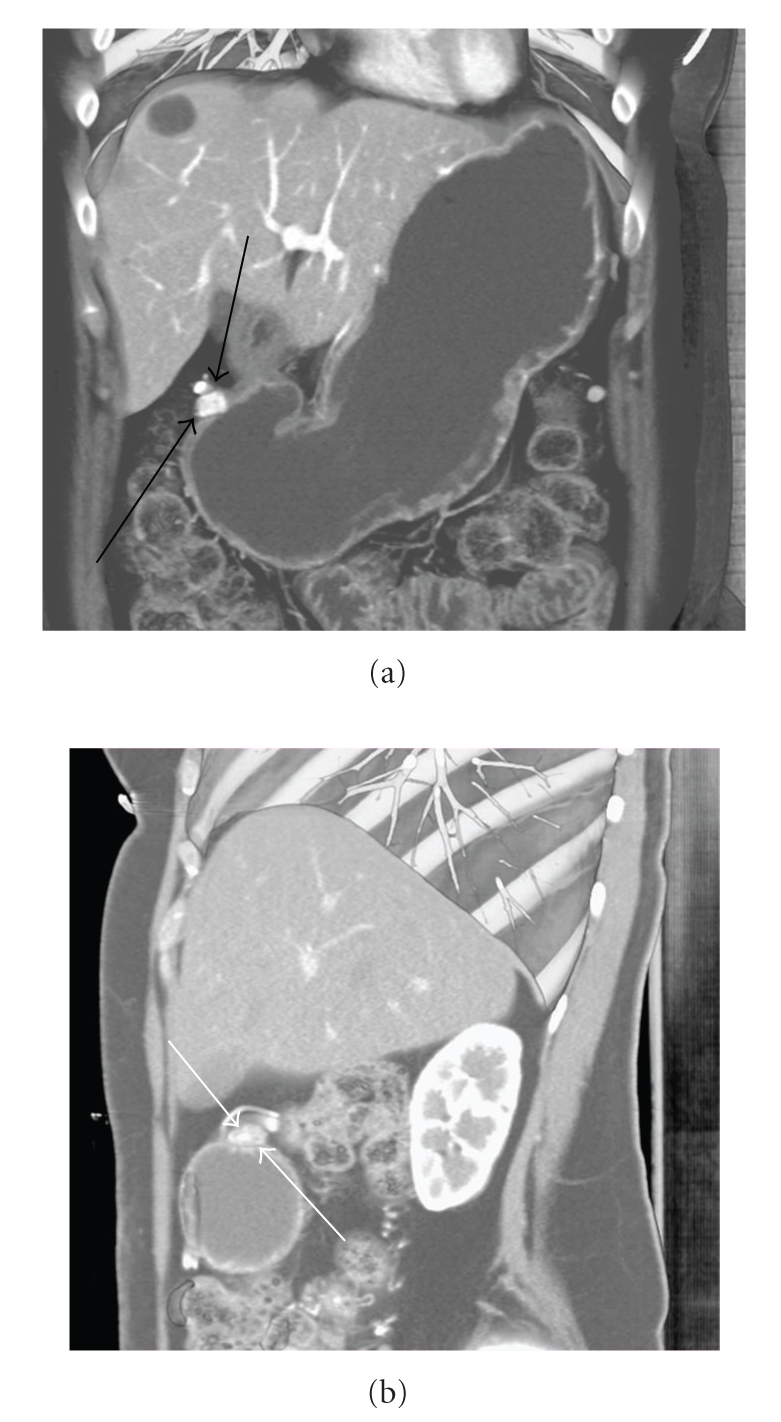
Volume rendering contrast enhanced MDCT with water as oral contrast. (a) Coronal and (b) Sagittal 3D images demonstrate a 1.7 cm densely enhancing mass along the gastric antrum (arrows).

**Figure 2:**
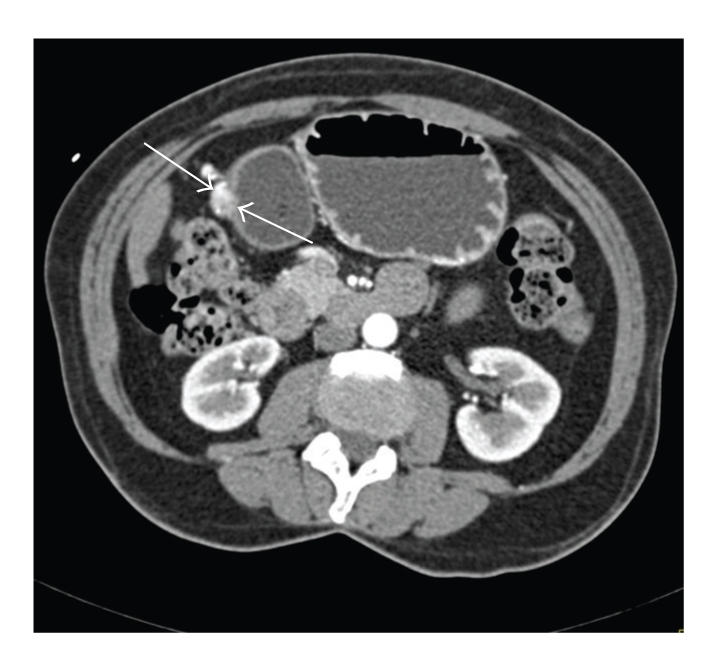
Standard axial image from the contrast enhance MDCT does show the antral lesion in retrospect (arrow). However, the radiologist did not note this on the initial interpretation.

**Figure 3:**
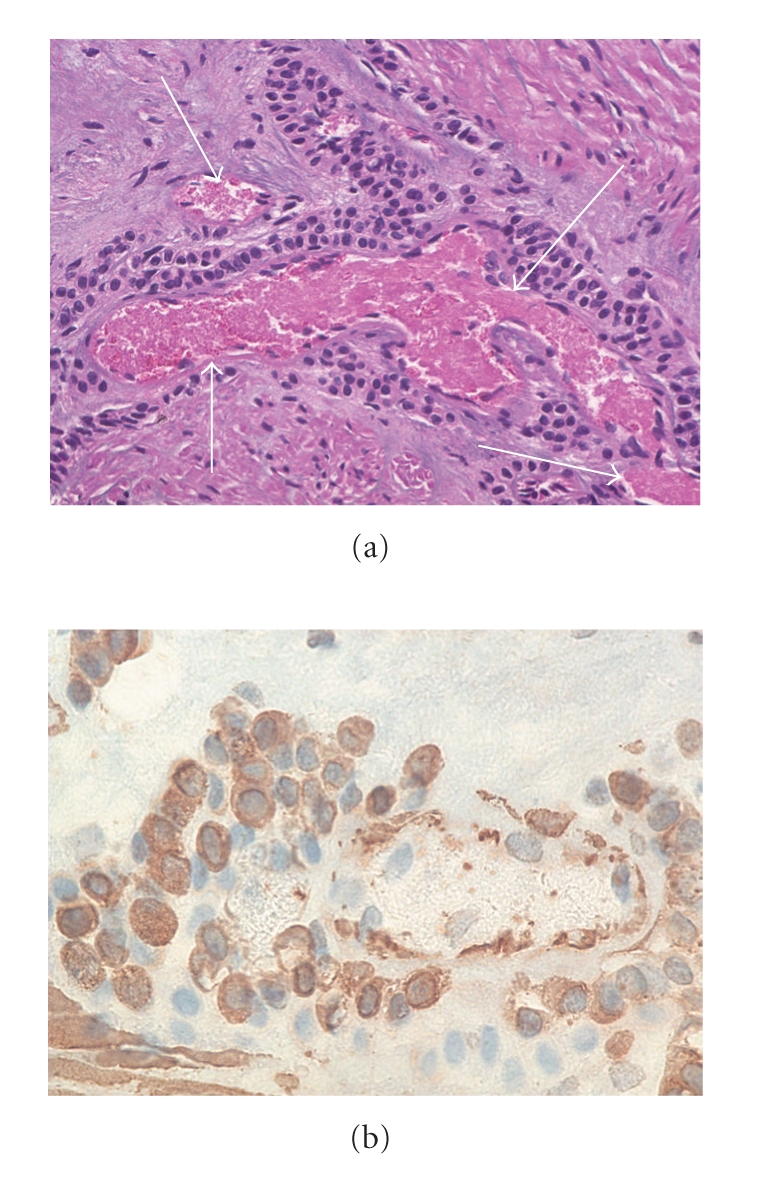
(a) High-magnification hematoxylin (×100) and eosin stained section that demonstrates a fragment of smooth muscle containing infiltrating uniform cells arranged around blood vessels (arrows) and (b) positive immunohistochemical stain for actin which are supportive of a diagnosis of gastric glomus tumor.

Initial interpretation of axial CT dataset did not report a gastric mass, presumedly related to the size of the lesion. Multiplanar reconstructions, however, did reveal a 1.7 cm well marginated solitary markedly enhancing mass in the gastric antrum (Figure 1). Retrospectively, the mass was found on axial CT images to display dense homogeneous enhancement in the arterial phase which persisted to the delayed phase (Figure 2). Portal vein and SMV were patent. SMA and celiac axis were unremarkable. No adenopathy was identified. Biopsy results reported a glomus tumor of the stomach (Figure 3).

## 3. Discussion

Glomus tumors, or glomangiomas, can occur anywhere within the gastrointenstinal tract, including the cecum. They, however, have been shown to occur most commonly in the first part of the gastric antrum [3]. Glomangiomas are submucosal masses that can remain asymptomatic and can be discovered incidentally or they may grow large enough to ulcerate the overlying mucosa and cause upper GI bleeding [1]. In a study of 32 gastrointestinal glomus tumors by Miettinen et al., these intramural neoplasms were found to range in size from 1.1 to 7 cm (median 2 cm), have a female predominance (23 females and 9 males), and were discovered mainly in late adulthood (median age 55 years old) [3]. 

Glomus tumors are soft in consistency and tend to change in size and shape with compression and peristalsis. This makes accurate diagnosis by either esophagogastrodeudonoscopy (EGD) or upper GI series challenging and nonspecific [1]. For this reason, axial CT images are an effective tool in the evaluation of these gastric masses. Aslo, given the fact that they are often small, the use of multiplanar and 3D imaging is valuable to visualize these tumors. Standard axial imaging may not be adequate, especially given the complex anatomy of the stomach in the axial plane. Certain areas, the gastric antrum for, example, can be potentially “blind” spots on axial images even to experienced radiologists.

The CT appearance of gastric glomus tumors is described in the literature as demonstrating dense homogenous enhancement in the arterial phase with prolonged enhancement in the delayed phase [4–6]. Glomus tumors are composed of various sized blood vessels lined by normal endothelial cells, which are surrounded by round glomus cells. It is hypothesized that these bulky vascular channels are responsible for the dense contrast enhancement, which often can be similar to the portal vein, IVC, or even descending aorta. This enhancement pattern can help distinguish glomus tumors from other submucosal lesions, such as leiomyomas, lipomas, and fibromas, which are not nearly as vascular [1]. 

In most of the cases reported, the tumor presents as a benign appearing gastric antral mass. Differential diagnosis considerations for hypervascular tumors in the stomach include similar appearing lesions such as gastrointestinal stromal tumor (GIST), arteriovenous (AV) malformations, carcinoid tumor, heterotopic pancreatic tissue, angioleiomyoma, and angiolipoma. These entities are difficult to differentiate based on clinical history and imaging findings alone, and in symptomatic patients, histopathologic evaluation is warranted. 

## 4. Conclusions

Glomus tumors of the stomach can have nonspecific imaging findings and a vague clinical presentation, making it challenging to both detect and differentiate from other disease entities. Given the potentially poor prognosis of some of these possible diagnoses, early detection and characterization is critical. Multiplanar reconstructions may be an effective adjunct in detecting gastric abnormalities that may elude diagnosis when axial MDCT sections are interpreted alone. As illustrated in this case, gastric glomus tumor should be considered when a hypervascular intramural gastric mass is detected on CT.
